# Myristicin regulates proliferation and apoptosis in oxidized low-density lipoprotein-stimulated human vascular smooth muscle cells and human umbilical vein endothelial cells by regulating the PI3K/Akt/NF-κB signalling pathway

**DOI:** 10.1080/13880209.2021.2010775

**Published:** 2021-12-14

**Authors:** Liang Luo, Huiying Liang, Luoying Liu

**Affiliations:** Department of Cardiology, Ganzhou People’s Hospital, Ganzhou, Jiangxi, P.R. China

**Keywords:** Coronary heart disease, atherosclerosis, migration, inflammation

## Abstract

**Context:**

Atherosclerosis (AS) is a chronic inflammatory disease. Human vascular smooth muscle cell (hVSMC) accumulation and human umbilical vein endothelial cell (HUVEC) dysfunction are associated with the pathogenesis of AS. This study explores whether myristicin plays a protective role in AS.

**Materials and methods:**

hVSMCs and HUVECs were stimulated with 100 μg/mL oxidized low-density lipoprotein (ox-LDL) to establish a cellular model of AS. Cell viability, lactate dehydrogenase (LDH) release and cell apoptosis were evaluated using MTT, LDH and flow cytometry assays, respectively. Cell migration and inflammatory cytokine release were assessed using Transwell assay and ELISA.

**Results:**

Myristicin (5, 10, 25, and 50 μM) had no obvious effect on cell viability or the activity of LDH in hVSMCs, while 100 and 200 μM myristicin markedly suppressed hVSMCs viability and increased LDH release. Myristicin had no obvious effect on cell viability or the activity of LDH in HUVECs. Myristicin inhibited viability and increased apoptosis in ox-LDL-treated hVSMCs, but was associated with increased proliferation and inhibited apoptosis in HUVECs stimulated by ox-LDL. Additionally, myristicin markedly suppressed ox-LDL-induced hVSMCs migration and the release of inflammatory cytokines, including MCP-1, IL-6, VCAM-1 and ICAM-1, in HUVECs. Results also demonstrated that the promoting effects of ox-LDL on the PI3K/Akt and NF-κB signalling pathway in both hVSMCs and HUVECs were abolished by treatment with myristicin.

**Discussion and conclusions:**

Myristicin regulated proliferation and apoptosis by regulating the PI3K/Akt/NF-κB signalling pathway in ox-LDL-stimulated hVSMCs and HUVECs. Thus, myristicin may be used as a new potential drug for AS treatment.

## Introduction

Coronary heart disease refers to heart disease caused by atherosclerosis (AS) of the coronary arteries, narrowing or occluding the vascular lumen, leading to myocardial ischaemia, hypoxia or necrosis. AS, the main cause of coronary heart and peripheral vascular disease, has been identified as a chronic inflammatory disease (Libby et al. 2019). AS is characterized by arterial intimal lesions, accumulation of complex carbohydrates, fibrous tissue hyperplasia and calcification, as well as arterial wall thickening and hardening (Feig et al. 2012; Gistera and Hansson 2017). Furthermore, previous reports have demonstrated that the accumulation of human vascular smooth muscle cells (hVSMCs) and dysfunction of human umbilical vein endothelial cells (HUVECs) are associated with the pathogenesis of AS. For example, Tao et al. (2019) revealed that long non-coding RNA (lncRNA) cancer susceptibility 11 improves AS by downregulating IL-9 and regulating VSMC apoptosis and proliferation. A study by Soltani et al. (2016) demonstrated that curcumin protects against ionizing radiation-induced AS by suppressing the adhesion of HUVECs to monocytes. Furthermore, oxidized low-density lipoprotein (ox-LDL) is considered to be the main marker of AS, and the accumulation of ox-LDL may lead to chronic inflammation, further accelerating the development of AS (Bian et al. 2020; Yang et al. 2020). However, the mechanism of ox-LDL in regulating the proliferation and apoptosis of hVSMCs and HUVECs requires further exploration.

In recent years, studies have demonstrated that the edible plant nutmeg has multiple pharmacological effects, such as hepatoprotective effects (Cao et al. 2020) and antidiabetic effects (Pashapoor et al. 2020). Myristicin, a natural compound in nutmeg, has been reported to have a good therapeutic effect in liver injury. Morita et al. (2003) suggested that myristicin can effectively prevent liver injury in a lipopolysaccharide/d-galactosamine-induced model. Furthermore, Martins et al. (2014) demonstrated that myristicin induced K562 leukaemia cell apoptosis via regulation of the mitochondrial pathway and DNA damage response pathways. However, the effect of myristicin on ox-LDL-induced cell apoptosis, as well as its mechanism in AS, needs to be further investigated.

Therefore, the present study was designed to illustrate the roles of myristicin in ox-LDL-induced hVSMCs and HUVECs during the pathogenesis of AS. In the present study, it was hypothesized that: i) hVSMCs and HUVECs induced by 100 μg/mL ox-LDL could promote cell injury and generate the AS model; ii) myristicin has a protective effect against AS in ox-LDL-stimulated hVSMCs and HUVECs; and iii) the latent mechanisms of the protective effects of myristicin may be associated with the regulation of the NF-κB and PI3K/Akt signalling pathways. In the present study, the biological functions of myristicin in ox-LDL-induced hVSMCs and HUVECs were analyzed. The present data demonstrated that myristicin markedly alleviated ox-LDL-induced hVSMC and HUVEC injury, as demonstrated by reduced hVSMC proliferation, induced apoptosis and inhibited migration. In addition, the present study demonstrated that myristicin affected ox-LDL-induced HUVEC proliferation, apoptosis and the expression of inflammatory cytokines. The potential regulatory network of the PI3K/Akt/NF-κB axis was also investigated in ox-LDL-treated hVSMCs and HUVECs. All these findings revealed that myristicin inhibited AS via inactivation of the PI3K/AKT/NF-κB signalling pathway and could be a novel therapeutic agent for AS.

## Materials and methods

### Cell culture and as a model establishment

hVSMCs and HUVECs were purchased from American Type Culture Collection, cultured in DMEM (Thermo Fisher Scientific, Inc.) supplemented with 10% FBS (Invitrogen; Thermo Fisher Scientific, Inc.) and 1% penicillin-streptomycin and maintained at 37 °C with 5% CO_2_ in an incubator. To establish the AS model *in vitro*, hVSMCs and HUVECs were stimulated with 100 μg/mL ox-LDL for 24 h (Wu et al. 2020).

To study the effects of myristicin on hVSMCs and HUVECs cells toxicity, hVSMCs and HUVECs were stimulated with various concentrations of myristicin (0, 5, 10, 25, 50, 100, 200 μM) at 37 °C for 24 h.

To study the effects of myristicin on ox-LDL-induced hVSMCs, hVSMCs were treated with 100 μg/mL ox-LDL at 37 °C for 24 h, followed by treatment with 5, 25 and 50 μM myristicin at 37 °C for another 24 h.

To study the effects of myristicin on ox-LDL-induced HUVECs, HUVECs were treated with 100 μg/mL ox-LDL at 37 °C for 24 h, followed by treatment with 5, 25 and 50 μM myristicin at 37 °C for another 24 h.

### MTT assay

hVSMCs and HUVECs were subjected to 100 μg/mL ox-LDL for 24 h, followed by myristin for another 24 h. After treatment, hVSMCs and HUVECs were seeded in 96-well plates (BD Biosciences) and cultured at 37 °C in a 5% CO_2_-containing incubator. Subsequently, cells were treated with 10 μL MTT (5 mg/mL) solution and continuously incubated for a further 4 h. After treatment, the solution was removed and 100 μL DMSO was added to each well to dissolve the formazan product. Finally, the optical density (OD) at a wavelength of 570 nm was measured using a multifunctional plate reader (BioTek Instruments, Inc.) after 15 min of vibration mixing according to the manufacturer's protocols.

### Lactate dehydrogenase (LDH) assay

The release of LDH was measured using an LDH-Cytotoxicity Assay Kit (Abcam). After treatment with 0, 5, 10, 25, 50, 100, and 200 μM myristin for 24 h, hVSMCs and HUVECs were seeded in 96-well plates. After specific treatment, the culture medium was collected to detect LDH activity. The absorbance was detected at 450 nm using a microplate reader (BioTek Instruments, Inc.).

### Flow cytometry analysis

hVSMCs and HUVECs were induced by 100 μg/mL ox-LDL for 24 h, followed by myristin for another 24 h. After treatment, hVSMC and HUVEC apoptosis were measured using an Annexin V-FITC/PI apoptosis detection kit (BD Biosciences) according to the manufacturer’s protocol. Finally, apoptotic cells were quantified using a flow cytometer (Becton, Dickinson and Company) and analyzed using CellQuest software.

### Reverse transcription-quantitative PCR (RT-qPCR)

After treatment, total RNA was extracted from hVSMCs and HUVECs using TRIzol^®^ reagent (Invitrogen; Thermo Fisher Scientific, Inc.) according to the manufacturer’s instructions. The miScript Reverse Transcription Kit (Qiagen, Inc.) was used to transcribe total RNA into cDNA. The ABI 7000 Real-Time PCR system (Applied Biosystems; Thermo Fisher Scientific, Inc.) and SYBR Green PCR Master Mix Kit (Takara Biotechnology Co., Ltd.) were used to examine the levels of MMP-9, Bcl-2, Bax, AKT, p65 and GAPDH. Primers were obtained from Sangon Biotech Co., Ltd. Primer sequences were listed as follows:

- MMP-9 forward 5′-TCGAACTTTGACAGCGACAAG-3′; Reverse 5′-TCAGTGAAGCGGTACATAGGGT-3′;
- Bax forward 5′-TCTGAGCAGATCATGAAGACAGG-3′; Reverse 5′-ATCCTCTGCAGCTCCATGTTAC-3′;
- Bcl-2 forward 5′-AGGATTGTGGCCTTCTTTGAG-3′; Reverse 5′-AGCCAGGAGAAATCAAACAGAG-3′;
- AKT forward 5′-GACAACTCAGGGGCTGAAGAGAT-3′; Reverse 5′-GGTCTGGAAAGAGTACTTCAGGG-3′;
- p65 forward 5′-ATGTGGAGATCATTGAGCAGC-3′; Reverse 5′-CCTGGTCCTGTGTAGCCATT-3′;
- GAPDH forward 5′-CATCATCCCTGCCTCTACTGG-3′; Reverse 5′-GTGGGTGTCGCTGTTGAAGTC-3′. Target gene expression levels were analyzed using the 2^–ΔΔCq^ method (Livak and Schmittgen 2001).

### Western blot analysis

Following treatment, total protein was extracted from hVSMCs and HUVECs using RIPA lysis buffer (Beyotime Institute of Biotechnology) and quantified using a BCA Protein Assay Kit (Invitrogen; Thermo Fisher Scientific, Inc.). Subsequently, averaged proteins were separated by 10% SDS-PAGE and transferred to a PVDF membrane. Following blocking with 5% skimmed milk in PBS with Tween-20 for 1.5 h, the membranes were incubated with primary antibodies against GAPDH (cat. no. 5174; dilution rate: 1:1,000; Cell Signalling Technology), MMP-9 (cat. no. 13667; dilution rate: 1:1,000; Cell Signalling Technology), Bcl-2 (cat. no. 4223; dilution rate: 1:1,000; Cell Signalling Technology), Bax (cat. no. 5023; dilution rate: 1:1,000; Cell Signalling Technology), phosphorylated (p-)AKT (cat. no. 4060; dilution rate: 1:1,000; Cell Signalling Technology), AKT (cat. no. 4685; dilution rate: 1:1,000; Cell Signalling Technology), p-p65 (cat. no. 3033; dilution rate: 1:1,000; Cell Signalling Technology) and p65 (cat. no. 8242; dilution rate: 1:1,000; Cell Signalling Technology) overnight at 4 °C. After washing in TBS with Tween-20, the membranes were incubated with corresponding secondary antibody (cat. no. 7074; dilution rate: 1:1,000; Cell Signalling Technology) for 1.5 h. Finally, proteins were assessed using ECL detection system reagents (Pierce; Thermo Fisher Scientific, Inc.) according to the manufacturer’s protocol. Densitometry was analyzed using ImageJ software (version 1.46; National Institutes of Health).

### Transwell migration assay

Following treatment, hVSMCs and HUVECs were harvested with trypsin (Gibco; Thermo Fisher Scientific, Inc.), and added to the top chamber of Co-star polycarbonate Transwells (Corning, Inc.), while DMEM with 10% FBS was added in the bottom chamber. Following culturing at 37 °C in a 5% CO_2_ atmosphere for 24 h, cells on the upper membrane surface were washed with cold PBS, fixed using 4% paraformaldehyde and stained with 0.1% crystal violet. Subsequently, the number of migratory cells on the membrane underside was counted in five random fields (Magnification: ×100) using an inverted microscope (TS100; Nikon Corporation).

### ELISA

After treatment, HUVECs were harvested and centrifuged for 10 min at 400 *g*. Subsequently, the levels of inflammatory factors [monocyte chemoattractant protein-1 (MCP-1), IL-6, vascular cell adhesion molecule 1 (VCAM-1) and intercellular adhesion molecule-1 (ICAM-1)] in HUVEC culture supernatant were detected using ELISA kits (BD Biosciences) according to the manufacturer’s instructions. The OD value of each well at 450 nm was measured using a Multiscan Spectrum (MD, USA) according to the manufacturer's instructions.

### Statistical analysis

Statistical analysis was performed using GraphPad Prism 6.0 software (GraphPad Software, Inc.). All results are presented as the mean ± SD of three independent experiments. Differences among groups were analyzed using Student’s *t*-test or one-way ANOVA followed by Tukey’s *post hoc* test. *p* < 0.05 was considered to indicate a statistically significant difference.

## Results

### Effects of myristicin on hVSMC and HUVEC toxicity

**Figure 1.**
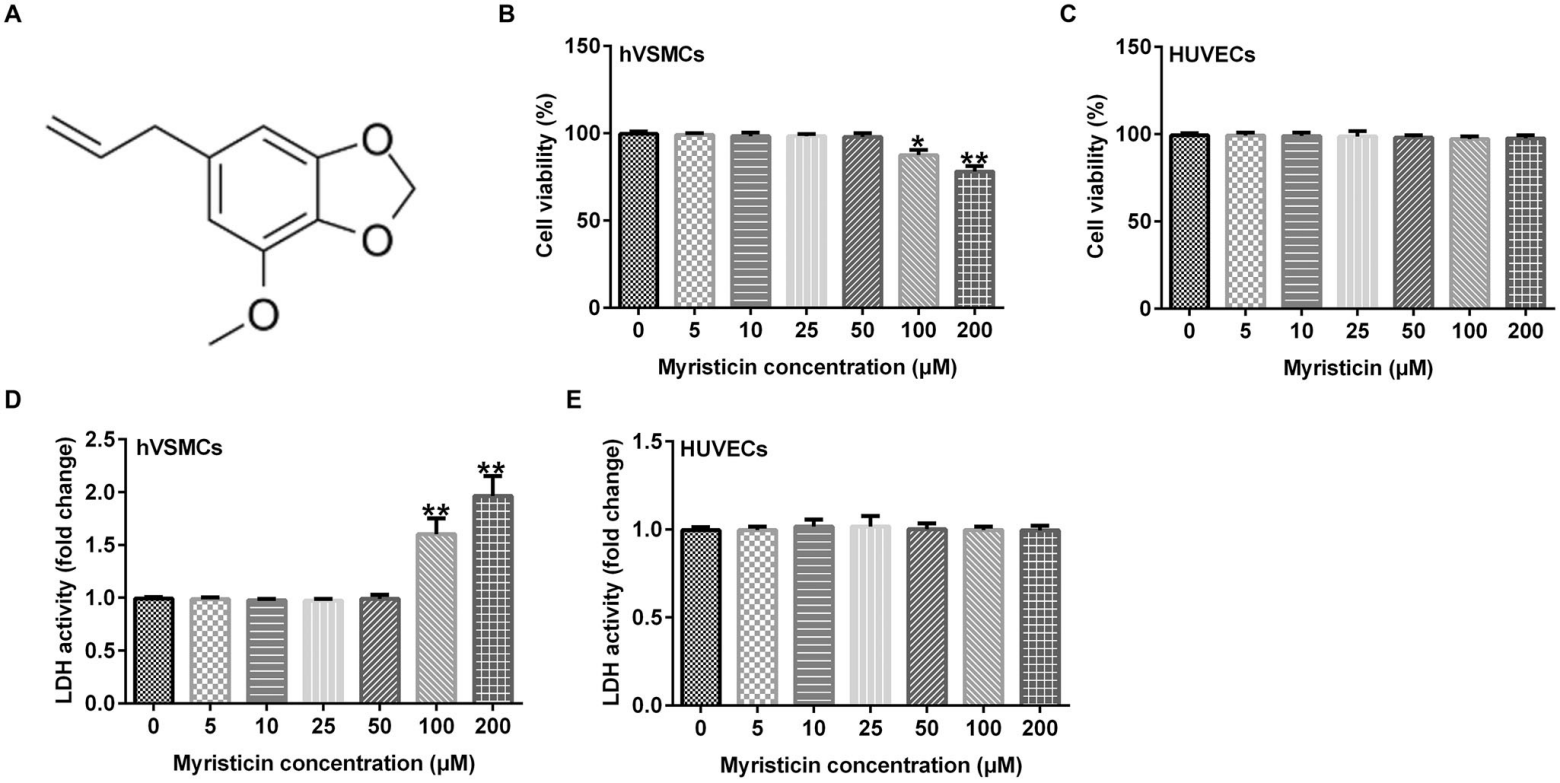
Myristicin had no distinct effects on hVSMC and HUVEC toxicity. A: Chemical formula of myristicin. Different concentrations of myristicin (0, 5, 10, 25, 50, 100, and 200 μM) were applied to induce hVSMCs and HUVECs for 24 h. (B and C) An MTT assay was performed to evaluate cell viability. (D and E) Detection of lactate dehydrogenase release. *p < 0.05, **p < 0.01 vs. control. HUVEC: human umbilical vein endothelial cell; hVSMC: human vascular smooth muscle cell.

Figure 1(A) shows the chemical formula of myristicin. First, the present study evaluated the effects of myristicin on hVSMC and HUVEC toxicity. hVSMCs and HUVECs were stimulated with various concentrations of myristicin (0, 5, 10, 25, 50, 100, and 200 μM) for 24 h. Subsequently, the role of myristicin in cell viability and LDH release was evaluated using MTT and LDH leakage assays, respectively. The present data revealed that there was no significant effect on hVSMC viability or LDH release following treatment with myristicin (5, 10, 25, and 50 μM; Figure 1(B–D)) compared with the control. Myristicin (100 and 200 μM) markedly inhibited hVSMC viability and enhanced LDH release. Furthermore, as shown in Figure 1(C–E), myristicin (0, 5, 10, 25, 50, 100, and 200 μM) exhibited no significant effects on viability and LDH activity in HUVECs. Therefore, 5, 25, and 50 μM myristicin were selected for subsequent experiments.

### Myristicin markedly reduces ox-LDL-induced hVSMC proliferation and apoptosis

**Figure 2.**
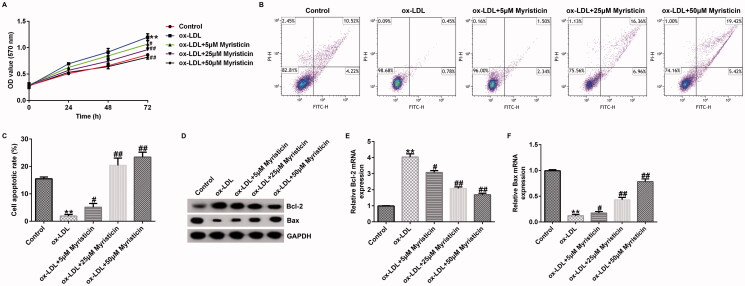
Myristicin regulates ox-LDL-induced hVSMC proliferation and apoptosis. hVSMCs were stimulated with 100 μg/mL ox-LDL for 24 h and subsequently induced by myristicin (5, 25, and 50 μM) for 24 h. (A) Cell proliferation was assessed using an MTT assay. (B) Flow cytometry analysis of cell apoptosis. (C) Quantification of apoptotic cells. (D) Determination of Bcl-2 and Bax protein expression. (E and F) Reverse transcription-quantitative PCR analysis of Bcl-2 and Bax. **p < 0.01 vs. control; ^#^*p* < 0.05, ^##^*p* < 0.01 vs. ox-LDL group. hVSMC: human vascular smooth muscle cell; ox-LDL: oxidized low-density lipoprotein.

To further illustrate the roles of myristicin in ox-LDL-induced hVSMCs, cells were subjected to 100 μg/mL ox-LDL for 24 h, followed by 5, 25, and 50 μM myristicin for another 24 h. Cell proliferation and apoptosis were evaluated using an MTT assay and flow cytometry. ox-LDL markedly promoted hVSMC proliferation (Figure 2(A)) and decreased the number of apoptotic cells (Figure 2(B,C)) compared with the control. Furthermore, cell apoptosis is usually regulated by apoptosis-specific genes, including Bcl-2 and Bax. The results of western blotting and RT-qPCR suggested that ox-LDL exposure markedly enhanced Bcl-2 expression (Figure 2(D)) and mRNA levels (Figure 2(E)), but reduced Bax expression (Figure 2(D)) and mRNA levels (Figure 2(F)). However, all these results were reversed by myristicin.

### Myristicin markedly suppresses ox-LDL-induced hVSMC migration

**Figure 3.**
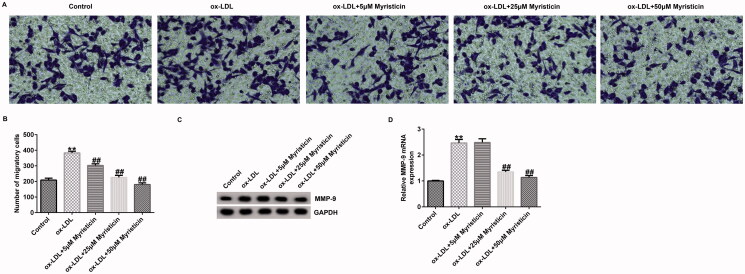
Myristicin inhibits ox-LDL-induced hVSMC migration. hVSMCs were stimulated with 100 μg/mL ox-LDL for 24 h and subsequently induced by myristicin (5, 25, and 50 μM) for 24 h. (A) Migration of hVSMCs. (B) Quantification of migratory cells. (C) Determination of MMP-9 protein expression. (D) Reverse transcription-quantitative PCR analysis of MMP-9. **p < 0.01 vs. control; ^##^*p* < 0.01 vs. ox-LDL group. hVSMC: human vascular smooth muscle cell; ox-LDL: oxidized low-density lipoprotein.

The present study also elucidated the role of myristicin in hVSMC proliferation. Cells were subjected to 100 μg/mL ox-LDL for 24 h, followed by 5, 25 and 50 μM myristicin for another 24 h. Subsequently, a Transwell assay was performed to evaluate hVSMC migration. As shown in Figure 3(A,B), exposure of hVSMCs to ox-LDL markedly enhanced cell migration, which was eliminated by myristicin. Furthermore, ox-LDL markedly enhanced MMP-9 protein and mRNA expression in hVSMCs, which was reversed by myristicin treatment (Figure 3(C,D)).

### Myristicin markedly reduces proliferation and apoptosis in ox-LDL-induced HUVECs

**Figure 4.**
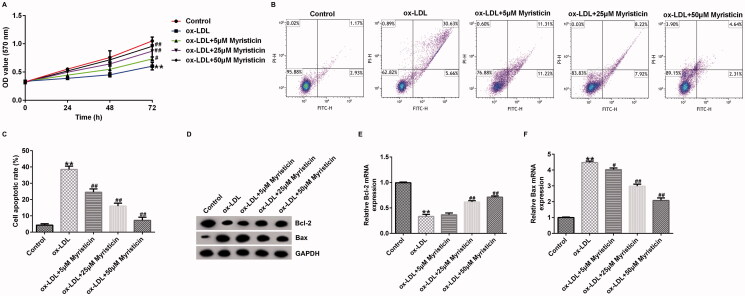
Myristicin regulates ox-LDL-induced HUVEC proliferation and apoptosis. HUVECs were stimulated with 100 μg/mL ox-LDL for 24 h and subsequently induced by myristicin (5, 25, and 50 μM) for 24 h. (A) Cell proliferation was assessed using an MTT assay. (B) Flow cytometry analysis of cell apoptosis. (C) Quantification of apoptotic cells. (D) Determination of Bcl-2 and Bax protein expression. (E and F) Reverse transcription-quantitative PCR analysis of Bcl-2 and Bax. **p < 0.01 vs. control; ^#^*p* < 0.05, ^##^*p* < 0.01 vs. ox-LDL group. HUVEC: human umbilical vein endothelial cell; ox-LDL: oxidized low-density lipoprotein.

To further investigate the regulatory mechanism of myristicin in ox-LDL-mediated AS, HUVECs were stimulated with 100 μg/mL ox-LDL for 24 h, followed by 5, 25, and 50 μM myristicin for another 24 h. The results of the MTT assay revealed that ox-LDL markedly inhibited HUVEC proliferation (Figure 4(A)), and flow cytometry analysis indicated that ox-LDL markedly promoted apoptosis in HUVECs (Figure 4(B,C)) compared with the control. Additionally, western blotting and RT-qPCR analysis revealed that Bax protein expression was enhanced (Figure 4(D,E)) but Bcl-2 expression was suppressed (Figure 4(D–F)) in HUVECs stimulated with ox-LDL, while these observations were the opposite in myristicin-treated HUVECs. Our aforementioned findings demonstrated the involvement of myristicin in AS.

### Myristicin reduces the ox-LDL-stimulated inflammatory response in HUVECs

**Figure 5.**
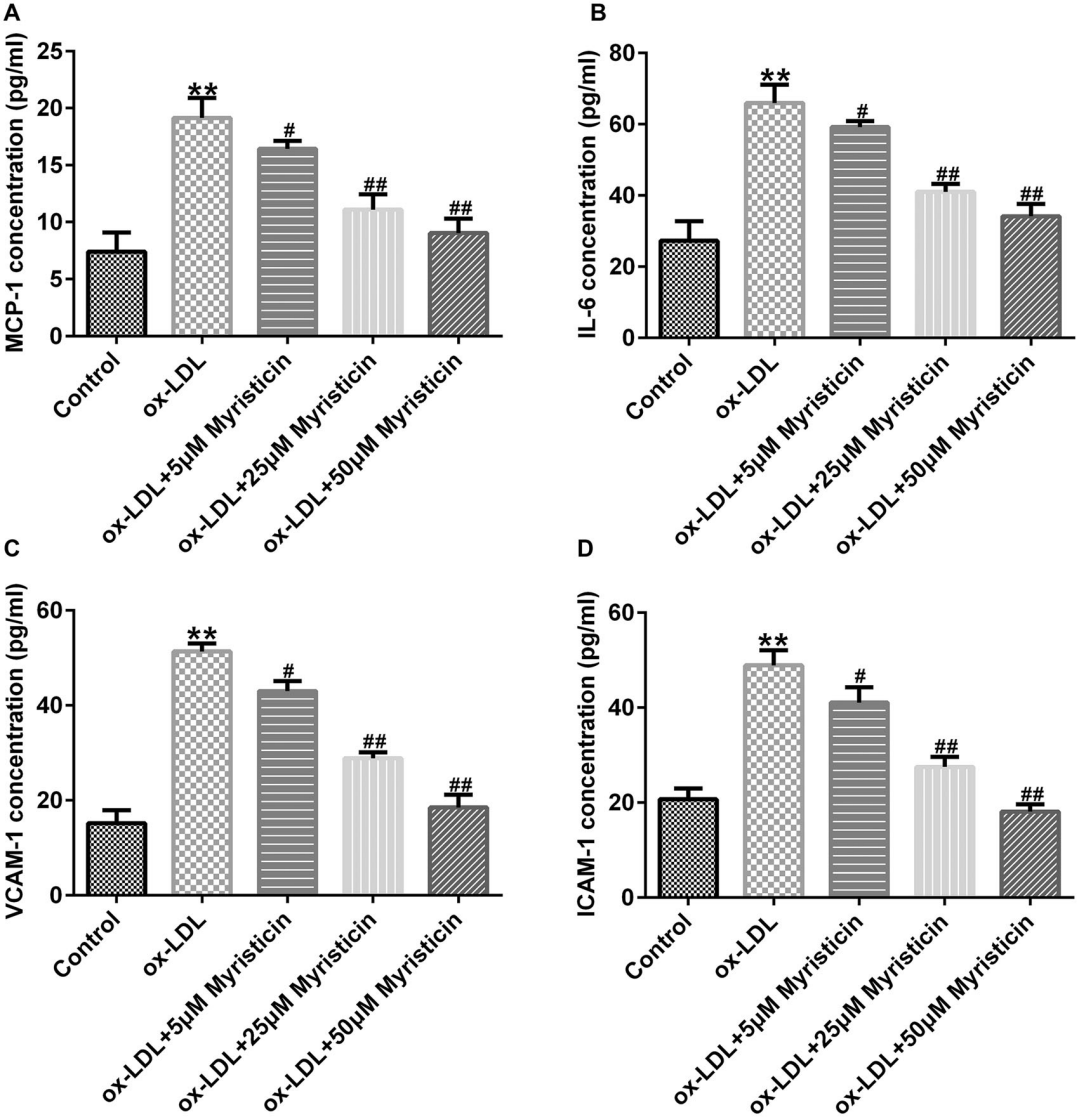
Myristicin inhibits ox-LDL-induced inflammatory cytokine secretion in HUVECs. HUVECs were stimulated with 100 μg/mL ox-LDL for 24 h and subsequently induced by myristicin (5, 25, and 50 μM) for 24 h. The excretion of inflammatory cytokines, including (A) monocyte chemoattractant protein-1, (B) IL-6, (C) vascular cell adhesion molecule 1 and (D) intercellular adhesion molecule-1, was analyzed using ELISA. **p < 0.01 vs. control; ^#^*p* < 0.05, ^##^*p* < 0.01 vs. ox-LDL group. HUVEC: human umbilical vein endothelial cell; ox-LDL: oxidized low-density lipoprotein.

Furthermore, the present study explored the effects of myristicin on the release of inflammatory cytokines, including MCP-1, IL-6, VCAM-1 and ICAM-1, in the supernatant of HUVECs. As shown in Figure 5(A–D), the secretion of MCP-1, IL-6, VCAM-1 and ICAM-1 was markedly promoted in ox-LDL-treated HUVECs, and myristicin markedly inhibited inflammatory secretion in ox-LDL-stimulated HUVECs. The present findings suggested that myristicin alleviated the ox-LDL-induced inflammatory response in HUVECs.

### Myristicin regulates the inactivation of the PI3K/Akt/NF-κB signalling pathway in ox-LDL-stimulated hVSMCs and HUVECs

**Figure 6.**
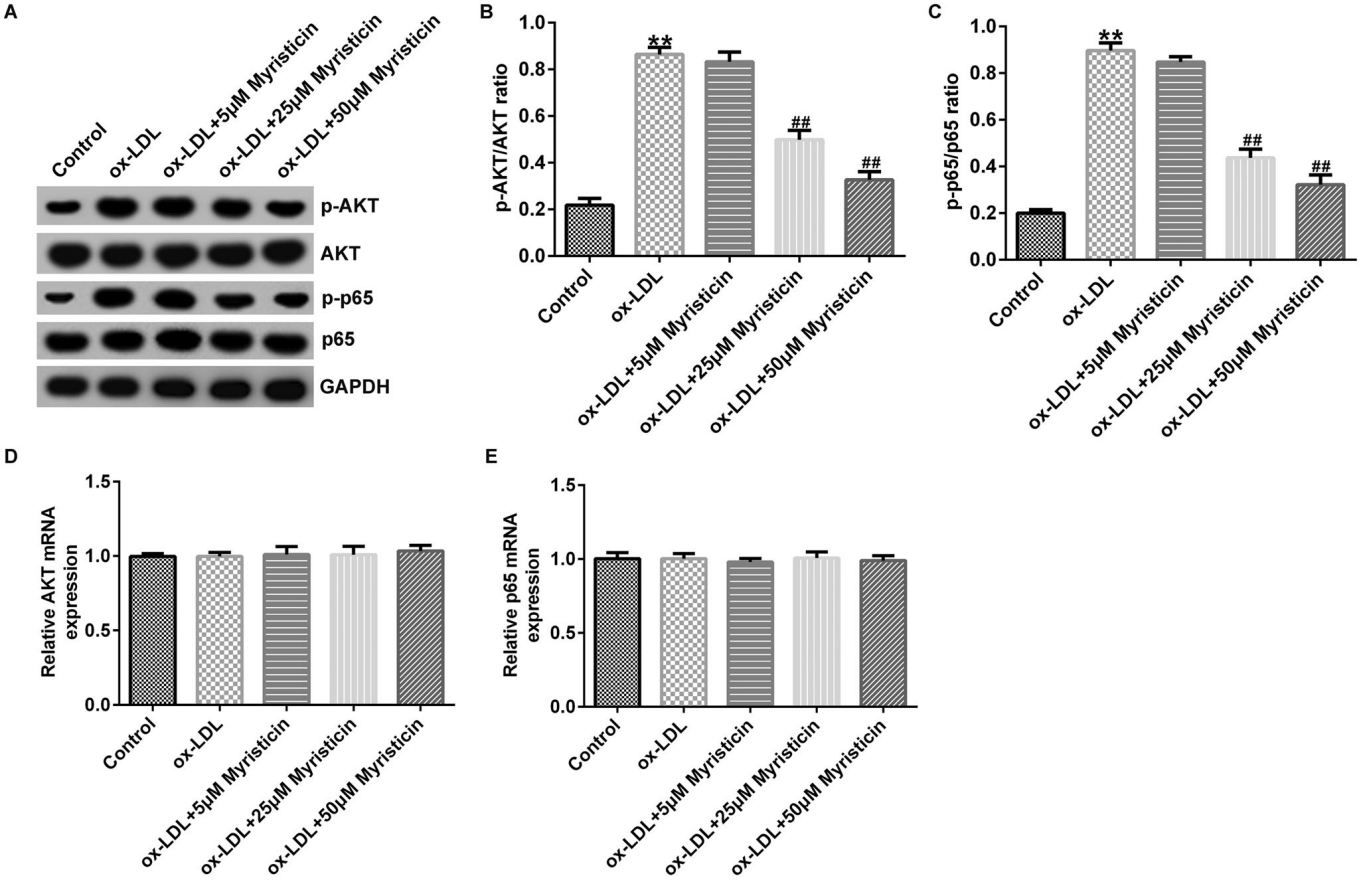
Myristicin inactivates PI3K/AKT and NF-κB signalling pathway in hVSMCs. hVSMCs were stimulated with 100 μg/mL ox-LDL for 24 h and subsequently induced by myristicin (5, 25, and 50 μM) for 24 h. (A) Protein levels of p-p65 and p-AKT were evaluated by western blotting. (B and C) Ratio of p-AKT/AKT and p-p65/p65. (D and E) Reverse transcription-quantitative PCR analysis of AKT and p65 in different groups. **p < 0.01 vs. control; ^##^*p* < 0.01 vs. ox-LDL group. hVSMC: human vascular smooth muscle cell; ox-LDL: oxidized low-density lipoprotein; p-: phosphorylated.

**Figure 7.**
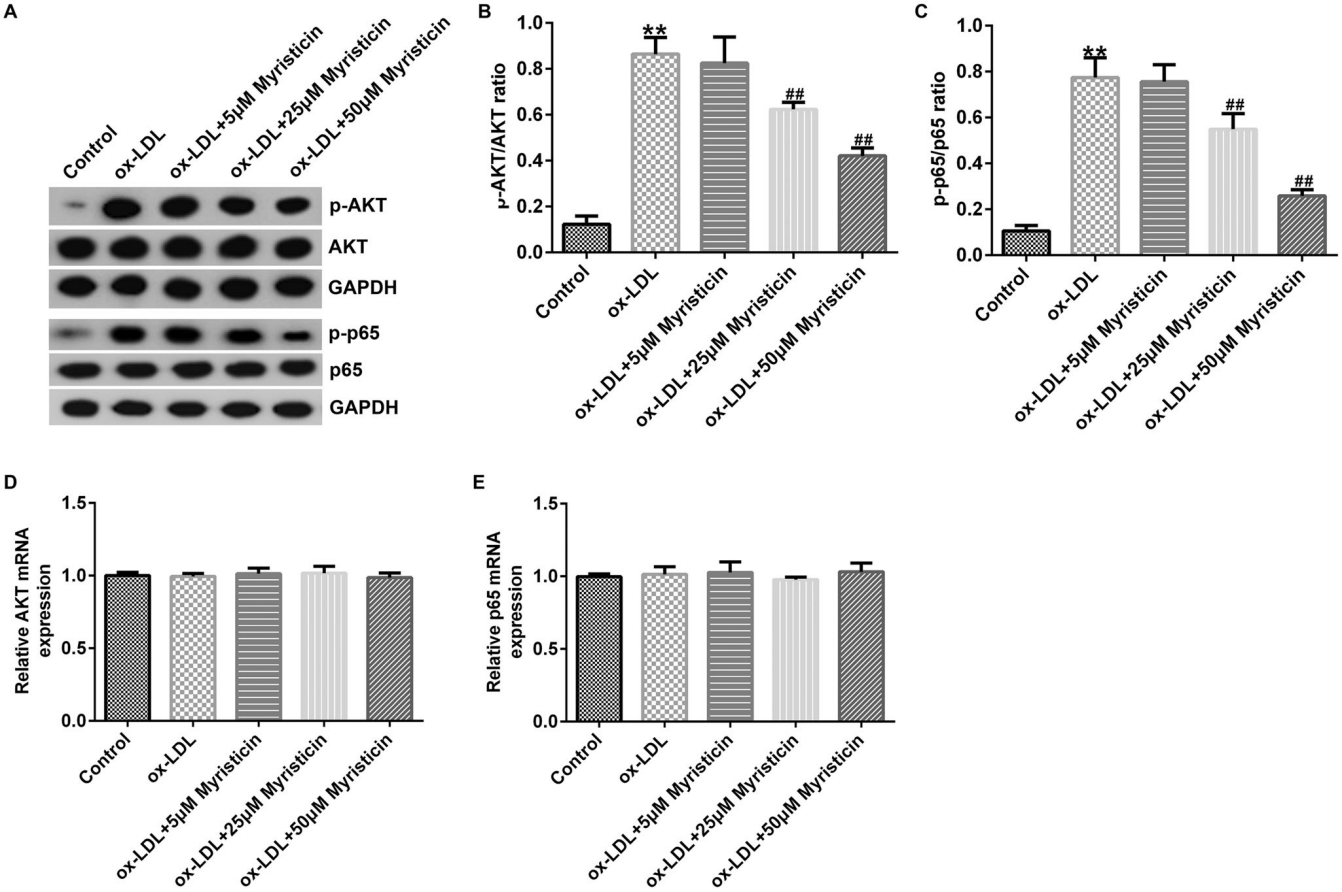
Myristicin inactivates PI3K/AKT and NF-κB signalling pathway in HUVECs. HUVECs were stimulated with 100 μg/mL ox-LDL for 24 h and subsequently induced by myristicin (5, 25, and 50 μM) for 24 h. (A) Protein levels of p-p65 and p-AKT were evaluated by western blotting. (B and C) Ratio of p-AKT/AKT and p-p65/p65. (D and E) Reverse transcription-quantitative PCR analysis of AKT and p65 in different groups. **p < 0.01 vs. control; ^##^*p* < 0.01 vs. ox-LDL group. HUVEC: human umbilical vein endothelial cell; ox-LDL: oxidized low-density lipoprotein; p-: phosphorylated.

Finally, to illustrate the possible mechanism of myristicin in AS, the PI3K/AKT and NF-κB signalling pathways were examined using western blotting and RT-qPCR. The present data demonstrated that ox-LDL markedly enhanced the levels of p-AKT and p-p65 (Figures 6(A) and 7(A)). In addition, the p-AKT/AKT (Figures 6(B) and 7(B)) and p-p65/p65 (Figures 6(C) and 7(C)) ratios were increased in ox-LDL-induced hVSMCs and HUVECs, and these effects were inhibited by myristicin. However, the mRNA expression levels of AKT (Figures 6(D) and 7(D)) and p65 (Figures 6(E) and 7(E)) exhibited no obvious changes. Overall, these data indicated that myristicin served a protective role against ox-LDL-stimulated AS by regulating the PI3K/Akt/NF-κB signalling pathway.

## Discussion

AS is a chronic inflammatory disease that is a major cause of numerous cardiovascular diseases, including heart attack (Keller et al. 2008), peripheral arterial disease (Fashanu et al. 2019), endothelial cell dysfunction (Berenji et al. 2020), diabetes mellitus (Colaiori et al. 2019) and cerebral infarction (Liu et al. 2017). Although great progress has been made in the pathogenesis and clinical research of AS, the therapeutic and treatment options are far from satisfactory, with AS still posing a major threat to human health worldwide. Increasing evidence has demonstrated that hVSMCs are induced by various pathogenic risk factors, including ox-LDL, exhibiting aberrant proliferation, apoptosis and migration during AS progression and development (Lu et al. 2021; Li et al. 2013). Furthermore, HUVEC injury is a key factor promoting AS progression (Hu et al. 2019), while the mechanism remains unclear.

Myristicin is the main component of nutmeg seeds and has been demonstrated to possess numerous beneficial properties (Morita et al. 2003; Martins et al. 2014). However, to the best of our knowledge, no studies have yet investigated the effects of myristicin on AS. Therefore, the present study was designed to investigate whether myristicin was associated with AS progression and to analyze the specific mechanisms involved. First, hVSMCs and HUVECs were stimulated with various concentrations of myristicin (0, 5, 10, 25, 50, 100, and 200 μM) for 24 h. The results of MTT and LDH assays revealed that cell viability and LDH release exhibited no obvious changes after 0, 5, 10, 25, and 50 μM myristicin treatment, indicating that low concentrations of myristicin did not lead to toxicity with hVSMC and HUVEC. Furthermore, 100 and 200 μM myristicin markedly inhibited hVSMC and HUVEC viability, and promoted LDH release, suggesting that it had a dose-dependent cytotoxic effect. Therefore, 5, 25, and 50 μM myristicin were selected for subsequent experiments.

Previous reports have demonstrated that ox-LDL is a main pro-inflammatory element in AS development, and hVSMCs and HUVECs induced by ox-LDL are widely applied to establish an AS model *in vitro* (Bian et al. 2020). To verify the cellular model of AS in the present study, 100 μg/mL ox-LDL was used to treat hVSMCs and HUVECs for 24 h to determine the association between myristicin and AS. Therefore, the present study sought to examine the effects of myristicin on proliferation and apoptosis in ox-LDL-stimulated hVSMCs. It was revealed that myristicin markedly decreased the viability of hVSMCs and enhanced apoptotic cells exposed to ox-LDL, indicating that the cellular model of AS was corroborated. Various apoptosis-associated proteins, such as Bcl-2 and Bax, are involved in the regulation of apoptosis. Bcl-2 has been identified as an anti-apoptotic marker and Bax as a pro-apoptotic marker, and disruption in the Bcl-2/Bax balance may lead to cell apoptosis, which results in AS development (Hu et al. 2007). Therefore, the present study subsequently determined the expression levels of Bcl-2 and Bax in ox-LDL-stimulated hVSMCs. The results of RT-qPCR and western blotting revealed that myristicin reversed the effects of ox-LDL on apoptosis-related gene expression, as demonstrated by ox-LDL reduced Bcl-2 levels and enhanced Bax expression. Cell migration is a key characteristic of vasculogenesis and endovascular repair. Zhang et al. (2020) reported that circular RNA circ_0003204 suppresses endothelial cell proliferation, migration and tube formation in AS via the microRNA-370-3p/TGFβ receptor 2/phosph-SMAD3 axis. Additionally, it was revealed that myristicin inhibited the effects of ox-LDL on the migration of hVSMCs. The present findings indicated that myristicin regulated ox-LDL-stimulated proliferation, apoptosis and migration in hVSMCs and may serve as a promising agent for preventing AS development. Consistent with previous studies, the present study also revealed that myristicin increased HUVEC proliferation, inhibited apoptosis, enhanced Bcl-2 expression and reduced Bax expression. Previous studies have demonstrated that dyslipidemia promotes the inflammatory response in AS. Wang et al. (2020) demonstrated that fargesin alleviates AS by accelerating reverse cholesterol transport and inhibiting the inflammatory response. Furthermore, the present study determined the effects of myristicin on the release of inflammatory cytokines, including MCP-1, IL-6, VCAM-1 and ICAM-1, in the supernatant of HUVECs. The present results indicated that the secretion of MCP-1, IL-6, VCAM-1 and ICAM-1 was markedly promoted in ox-LDL-treated HUVECs, and myristicin markedly inhibited inflammatory secretion in ox-LDL-stimulated HUVECs. The present findings suggested that myristicin alleviated the ox-LDL-induced inflammatory response in HUVECs.

Numerous studies have demonstrated that multiple pathways are involved in cellular apoptosis, including the nuclear translocation of the NF-κB p65 signalling pathway and the PI3K/AKT signalling pathway. Fu et al. (2019) suggested that scutellarin exerts protective effects against AS in rats by regulating the Hippo-FOXO3A and PI3K/AKT signalling pathways. The results of Simion et al. (2020) revealed that lncRNA vascular inflammation and AS lncRNA sequence regulate AS by regulating the NF-κB and MAPK signalling pathway. To better understand the underlying mechanisms of myristicin in the progression of AS, the levels of p-AKT, AKT, p-p65 and p65 were determined. The present results demonstrated that ox-LDL markedly increased the levels of p-AKT and p-p65. In addition, the ratios of p-AKT/AKT and p-p65/p65 were increased in ox-LDL-induced hVSMCs and HUVECs, and these were inhibited by myristicin. However, the mRNA levels of p65 and AKT exhibited no obvious changes. Overall, the present data indicated that myristicin served a protective role in ox-LDL-stimulated cell apoptosis via regulation of the PI3K/Akt/NF-κB signalling pathway.

## Conclusions

The present study suggested that myristicin suppressed the ox-LDL-stimulated inflammatory response by regulating the PI3K/Akt/NF-κB signalling pathway in AS progression. Therefore, myristicin may be a promising therapeutic target for ox-LDL-stimulated immune inflammation in AS. The present report provided potential useful therapeutic targets for AS. However, this study is only a preliminary *in vitro* study of the effect of myristicin on AS. In order to make myristicin’s role in AS more convincing, *in vivo* experiments are needed. We will further study the functions and mechanisms of myristicin in animal models of AS in the future.

## Authors’ contribution

Liang Lu and Huiying Liang contributed to study design, data collection, statistical analysis, data interpretation and manuscript preparation. Luoying Liu contributed to statistical analysis and manuscript preparation. All authors read and approved the final manuscript.

## Data Availability

The datasets used and/or analyzed during the current study are available from the corresponding author on reasonable request.
